# Effects of psychedelic microdosing versus conventional ADHD medication use on emotion regulation, empathy, and ADHD symptoms in adults with severe ADHD symptoms: A naturalistic prospective comparison study

**DOI:** 10.1192/j.eurpsy.2024.8

**Published:** 2024-02-14

**Authors:** Eline C.H.M. Haijen, Petra P.M. Hurks, Kim P.C. Kuypers

**Affiliations:** Department of Neuropsychology and Psychopharmacology, Faculty of Psychology and Neuroscience, Maastricht University, Maastricht, The Netherlands

**Keywords:** ADHD, emotion regulation, empathy, microdosing, psychedelics

## Abstract

Adults with attention-deficit hyperactivity disorder (ADHD) often struggle with emotion regulation (ER), impacting their empathic skills and relationships. ADHD medication might not be as effective for ER issues as for ADHD symptoms. Microdosing (MD) psychedelics has shown promise for ADHD treatment and previous studies reported social-emotional benefits. Two online prospective studies investigated MD effects on ER and empathy in adults with severe ADHD symptoms across three assessments: baseline, two-, and four-week post-initiation. Study 1 examined adults initiating MD on their own (n = 233, n = 64, and n = 44) and found positive effects on ER (cognitive reappraisal and expressive suppression) and aspects of empathy (perspective-taking and personal distress). Study 2, including a control group and an ADHD symptom scale, compared individuals only MD (n = 180, n = 50, and n = 38) to individuals using conventional ADHD medication (n = 37, n = 27, and n = 28). After 4 weeks, ADHD symptoms were lower in the MD group. Only improvements in expressive suppression persisted after adding the control group. This study indicates the positive effects of MD psychedelics on ADHD symptoms and ER in adults with severe ADHD symptoms while lacking evidence for effects on empathy.

## Introduction

Attention–deficit hyperactivity disorder (ADHD) is characterized by age-inappropriate levels of inattention, hyperactivity, and/or impulsivity [1, 2]. Next to these core symptoms, deficits in emotion regulation (ER) are considered problematic in ADHD [3–5]. ER involves the ability to reinterpret an emotion-eliciting situation (i.e., cognitive reappraisal [CR]) and to inhibit strong positive and negative emotional responses (i.e., expressive suppression [ES]) [6]. Previous studies have related ER to empathy, suggesting a moderating role of ER on empathy [7, 8], vice versa [9], or proposed an integrated framework of both [10]. Emotional empathy involves sharing others’ emotions, while cognitive empathy involves recognizing and understanding others’ thoughts and feelings. Research focusing on empathy in adults with ADHD is sparse, but some studies have shown impaired emotional [11, 12] and cognitive [13] empathy skills in adults with ADHD. Deficits in ER and/or empathy may cause inappropriate responses in emotional and social situations, creating obstacles to keeping healthy interpersonal relationships [14, 15].

Earlier studies have shown that standard ADHD medications, such as amphetamines, methylphenidate, and atomoxetine, effectively address ER impairments, but to a lesser degree than core ADHD symptoms [16–18]. A recent review proposed that conventional ADHD medication could improve empathy [19]. However, only 1 out of 17 studies included in this systematic review focused on adults with ADHD, the other studies focused on ADHD-diagnosed children. ADHD medication may inadequately target non-core symptoms like emotional and social functioning in adults, necessitating exploration of alternative treatments [20].

Individuals diagnosed with severe ADHD symptoms reported self-treating their symptoms using low, repeated doses of psychedelic substances (“microdosing” [MD]), such as psilocybin or lysergic acid diethylamide (LSD) [21–24]. MD was deemed more effective than conventional ADHD treatments [23] and correlated with discontinuation of various prescribed psychiatric medications, as indicated by retrospective survey studies [25]. While microdoses of psychedelics have been proposed to enhance emotional and social functioning in general population samples [22, 24, 26–31], no MD study to date has specifically investigated ER and/or empathy. Most findings are based on naturalistic studies, though one placebo-controlled study in healthy adults demonstrated acute changes in brain circuits associated with emotional processing after low-dose LSD administration [32]. In contrast, one study including self-administration of microdoses of psilocybin did not find improvements in emotional processing in healthy adults [33]. The effects of MD on ER and empathy in adults with severe ADHD symptoms remain unexplored.

Study 1 aimed to investigate the baseline changes in ER and empathy after 4 weeks of MD in individuals diagnosed with ADHD diagnosis and those without an official diagnosis but experiencing severe ADHD symptoms, using a prospective naturalistic design. We included individuals without an ADHD diagnosis, due to frequent misdiagnosis or lack of recognition, despite experiencing significant challenges [15, 34]. A previous study showed poorer outcomes in adults with symptomatic ADHD lacking a diagnosis [35], emphasizing the importance of studying this population. Participants without an official diagnosis were therefore treated equally in our study, based on their baseline ADHD symptom severity. The hypothesis posited ER and empathy improvements after 4 weeks of MD compared to baseline, without specifying what aspects of ER (i.e., CR and/or ES) and empathy (cognitive and/or emotional) would improve. Finally, we examined the effects of conventional medication use, and comorbid diagnoses on the change in ER and empathy over time.

Study 2 aimed to compare ADHD symptom severity, ER, and empathy across three time points in the MD group (not using conventional ADHD medication) and a group using conventional medication (not engaging in MD), contextualizing Study 1’s findings. We expected the MD group to report worse ADHD symptom severity at baseline, but to show improved ADHD symptom severity, ER, and empathy after 4 weeks compared to the conventional ADHD medication group.

The current studies’ overarching goal is to explore the practices and self-reports of adults with severe ADHD symptoms that self-medicate with MD, paving the way for potential investigation of MD for ADHD in a randomized controlled trial, potentially expanding ADHD treatment options.

## Study 1

### Methods

#### Study design and participants

The current study was part of a larger online prospective naturalistic study [21] and aimed to measure ER and empathy at baseline, before MD initiation, and 2 and 4 weeks after MD initiation in an ADHD population. All participants had the intention to microdose on their own initiative. The study involved no experimental manipulations, but only collected data during participants’ self-initiated MD practices. Adults with diagnosed ADHD and those without a diagnosis but experiencing severe symptoms were invited to participate. ADHD symptom severity was determined at baseline using the short, screening version of the Conners’ Adult ADHD Rating Scale (CAARS-S:SV) [36]. Individuals without an ADHD diagnosis with T-scores below 65 on each subscale were excluded since this was indicative of not experiencing severe ADHD symptoms. All participants provided informed consent before study initiation.

#### Study procedure

Participants were recruited online via www.microdosinginstitute.com, where an advertisement detailed the study’s purpose, procedures, and contact information. Individuals were requested to provide consent 1–3 days before MD-initiation and were then automatically directed to the baseline survey. Participants received links to follow-up surveys at 2 and 4 weeks post-baseline via email. Reminder emails were sent for incomplete surveys. Surveys took 15–20 min to complete. Information regarding MD substance and dose administered were collected through daily links until the 4-week time point. Data were collected between November 2020 and July 2021, with ethical approval received from the Ethics Review Committee of Psychology and Neuroscience at Maastricht University (ERCPN-215_05_11_2019_A1).

### Measures

#### Demographic information and history of substance use

Demographic information and history of psychedelic use were collected at baseline (Table 1).

**Table 1.** Demographic information collected at baseline for the sample at baseline and the 2- and 4-week time points

Time point		Baseline	2-week	4-week
*n*		233	64	44
Age (mean ± standard deviation)		35.3 ± 10.7	35.4 ± 10.1	37.2 ± 9.7
		*n* (%)	*n* (%)	*n* (%)
Biological sex	Male	116 (49.8)	30 (46.9)	21 (47.7)
	Female	117 (50.2)	34 (53.1)	23 (52.3)
Gender	Male	112 (48.1)	28 (43.8)	19 (43.2)
	Female	117 (50.2)	34 (54.5)	23 (52.3)
	Other	4 (1.7)	2 (3.0)	2 (4.5)
Continent of current residence	Europe	193 (82.8)	61 (95.3)	43 (97.7)
	America (North)	33 (14.2)	2 (3.1)	1 (2.3)
	America (South)	5 (2.1)	1 (1.6)	–
	Asia	2 (0.9)	–	–
	Africa	–	–	–
	Australia/Oceania	–	–	–
Highest level of education	Tertiary (university, trade school, college)	107 (73.0)	48 (75.0)	31 (70.5)
	Secondary (high school, academies, gymnasium, etc.)	54 (23.2)	15 (23.4)	13 (29.5)
	Primary (elementary)	9 (3.9)	1 (1.6)	–
Daily occupation	Computer/office work	57 (24.5)	16 (25.0)	12 (27.3)
	Studying	43 (18.5)	11 (17.2)	6 (13.6)
	Working with people	39 (16.7)	15 (23.4)	9 (20.5)
	Creative work	31 (13.3)	9 (14.1)	8 (18.2)
	Physical work	13 (5.6)	4 (6.3)	4 (9.1)
	Other	50 (21.5)	9 (14.1)	5 (11.4)
Currently diagnosed^a^	Yes	166 (71.2)	49 (76.6)	34 (77.3)
	No	67 (28.8)	15 (23.4)	10 (22.7)
Type of disorder^b^	ADHD	159 (68.2)	45 (70.3)	34 (72.3)
	Depression	44 (18.9)	7 (10.9)	5 (11.4)
	Anxiety disorder	39 (16.7)	8 (12.5)	6 (13.6)
	PTSD	17 (7.3)	4 (6.3)	1 (2.3)
	Personality disorder	11 (4.7)	3 (4.7)	–
	Dyslexia	12 (5.2)	2 (3.1)	2 (4.5)
	Migraines	8 (3.4)	3 (4.7)	3 (6.8)
	Chronic pain	7 (3.0)	2 (3.1)	2 (4.5)
	Cluster headaches	6 (2.6)	3 (4.7)	3 (6.8)
	Substance use disorder	4 (1.7)	1 (1.6)	–
	Autism/Asperger syndrome	4 (1.7)	1 (1.6)	–
	OCD	3 (1.3)	1 (1.6)	–
	Bipolar disorder	3 (1.3)	–	–
	Schizophrenia	–	–	–
	Other	23 (9.9)	6 (9.4)	3 (6.8)
Experience with psychedelic drug^c^	Yes	191 (82.0)	50 (78.1)	37 (78.7)
	No	42 (18.0)	14 (21.9)	10 (21.3)

Absolute and relative frequencies are shown. Numbers in parentheses indicate the percentage corresponding to the absolute frequencies.

a Are you currently diagnosed by a medical doctor or therapist with a psychiatric, neurological, or physical disorder?

b Numbers do not add up to the sample size, because multiple answers were possible.

c Do you have experience with at least one of the following psychedelics? Ayahuasca, DMT, 5-MeO-DMT, LSD, novel lysergamides (e.g., 1P-LSD, ALD-52), psilocybin/psilocin (magic mushrooms, truffles), *salvia divinorum*, ibogaine, mescaline (e.g., san pedro, peyote).

#### Psychiatric and physiological diagnoses

Information about current diagnoses of a psychiatric, neurological, and/or physical disorder from medical professionals was collected at baseline (Table 1). A “comorbidity” variable differentiated participants with and without comorbid diagnoses alongside ADHD (0 = only ADHD or no ADHD diagnosis, 1 = ADHD and at least one comorbid diagnosis).

ADHD-diagnosed participants provided their age at diagnosis and current medication details, if any. If ADHD medication was used in the past but not currently, reasons for discontinuation were asked (Table 2). A variable ‘medication use’ distinguished participants who were solely MD from those using ADHD medication alongside MD during the study (0 = only MD; 1 = MD and using ADHD medication).

**Table 2.** ADHD-related information collected at baseline from the subsample who had been diagnosed with ADHD in the past (*n* = 159)

		*n* (% of 159)
Age of receiving ADHD diagnosis	Younger than 10 years old	9 (5.7)
	10–14 years old	7 (4.4)
	15–19 years old	17 (10.7)
	20–29 years old	71 (44.7)
	30–39 years old	38 (23.9)
	40–49 years old	12 (7.5)
	Over 50 years old	5 (3.1)
Current ADHD medication use	No, never	22 (13.8)
	Used to, but not anymore	84 (52.8)
	Reasons for discontinuing	*n* (% of 84)
	It did not relieve my symptoms	17 (20.2)
	Because of psychological side-effects	51 (60.7)
	Because of physical side-effects	54 (63.1)
	I do not want to mention	1 (1.2)
	Other reasons	21 (25.0)
	Yes, I am currently using these	53 (33.3)
	Medication types	*n* (% of 53)
	Amphetamine	27 (50.9)
	Methylphenidate	21 (39.6)
	Other	5 (9.4)

#### MD substance and dose

Daily surveys during the 4-week study duration asked about microdose intake, substance, and dose (Table 3).

**Table 3.** Microdosing substances and doses used during the study

	Frequency (% of 117)	Mean dose (SD)
Psilocybin-containing mushrooms, truffles^a^	91 (77.8)	722 mg (485.5)
Novel lysergamides (e.g., 1P-LSD, ALD-52)	14 (12.0)	17.5 μg (31.1)
LSD	11 (9.5)	12 μg (6.4)
Ayahuasca	1 (0.9)	–

a No further data were collected on whether psilocybin-containing mushrooms/truffles were dried or fresh.

#### ADHD symptoms

The CAARS-S:SV assessed ADHD symptom severity at baseline [36]. The 30-item questionnaire consists of three subscales: inattention, hyperactivity/impulsivity, and ADHD index, with a DSM-IV ADHD total symptom score calculated by summing the inattention and hyperactivity/impulsivity subscale scores. The ADHD index measures problems related to ADHD that are not diagnostic criteria. Items were rated on a four-point Likert scale (0–3). Min–Max for inattention and hyperactivity/impulsivity were 0–27, for the ADHD index 0–36, and for the DSM-IV ADHD symptom total raw score 0–54. T-scores were calculated for all subscales, with scores of at least 65 indicating clinically elevated ADHD symptoms [36]. Participants without an ADHD diagnosis and T-scores below 65 on all subscales were excluded from the analyses.

#### Emotion regulation

The 10-item ER Questionnaire (ERQ) [6] assessed CR and ES at baseline, 2 weeks, and 4 weeks. CR involves changing the understanding of emotion-eliciting situations to experience and express more positive emotions. ES involves inhibiting emotion-expressive behavior and correlates negatively with life satisfaction. Items were rated on a seven-point Likert scale (1–7). Min–Max for CR were 0–42 and for ES 0–28. Higher CR and lower ES scores indicate better ER. The ERQ has good internal consistency and validity [37].

#### Empathy

The Interpersonal Reactivity Index (IRI) [38] measured empathy at baseline, 2-, and 4 weeks. Cognitive empathy was assessed through perspective-taking (PT) (i.e., adopting the psychological viewpoint of others) and fantasy (F) (i.e., transposing oneself imaginatively in the actions and feelings of fictitious characters described in books). Emotional empathy was measured via empathic concern (EC) (i.e., feeling sympathy and concern for others) and personal distress (PD) (i.e., self-directed anxiety and unease in tense interpersonal settings). PD negatively correlates with social functioning [39]. Items are rated on a five-point Likert scale (0–4), Min–Max for all subscales was 0–28. Higher scores on PT, EC, and F, and lower scores on PD, indicate greater empathy. The IRI is a widely used scale assessing a multidimensional view of trait empathy, with good psychometric properties [38].

### Statistical analyses

The data analysis was conducted using IBM SPSS Statistics version 26. Information regarding demographics, diagnoses, and drug types and doses used for MD were described using descriptive statistics. Linear mixed model (LMM) analyses assessed ER and empathy changes at 2 and 4 weeks post-MD versus baseline. Each LMM included time (baseline [0 W], 2- [2 W], and 4 weeks [4 W]) as within-subjects factor. Medication use (yes/no) and comorbidity (yes/no) were covariates in all LMMs. The fixed part of the models consisted of time, medication use, comorbidity, and the interaction terms between time and medication use, and time and comorbidity.

To assess MD-induced effects on ER, ERQ subscales (CR and ES) served as dependent variables in separate LMMs. Emotional and cognitive empathies were evaluated using the four IRI subscales (PT, EC, F, PD) in four distinct LMMs.

The best-fitting covariance structure was chosen based on the lowest Akaike’s information criterion (AIC) value. Restricted maximum-likelihood (REML) estimation addressed missing data. Pairwise comparisons between time points were corrected for multiple comparisons using Bonferroni correction. To correct for multiple testing, the significance level of. 05 was divided by the number of subscales per construct, resulting in a significance level of. 025. Effect sizes were described using partial eta squared (*η*_p_^2^) values, with 0.01, 0.09, and 0.25 considered small, medium, and large, respectively [40]. Only significant effects will be described.

## Results

Out of 356 individuals who consented and started the survey, 70% completed the baseline survey and received links to the subsequent surveys (*n* = 247). Fast responses (response time <50% of the median response time) were visually checked and led to two respondents being excluded due to unrealistic responses. Further, 12 respondents without an ADHD diagnosis were excluded due to T-scores lower than 65 on all CAARS-S:SV subscales. The final sample sizes for analyses were 233, 64, and 44 at 0 W, 2 W, and 4 W, respectively. Demographic information for the three time points is presented in Table 1, while ADHD-related information for the diagnosed subsample is in Table 2. MD substances and doses were provided by only half of the sample through daily reports and are given in Table 3.

### Emotion regulation

#### Cognitive reappraisal

**Figure 1.**
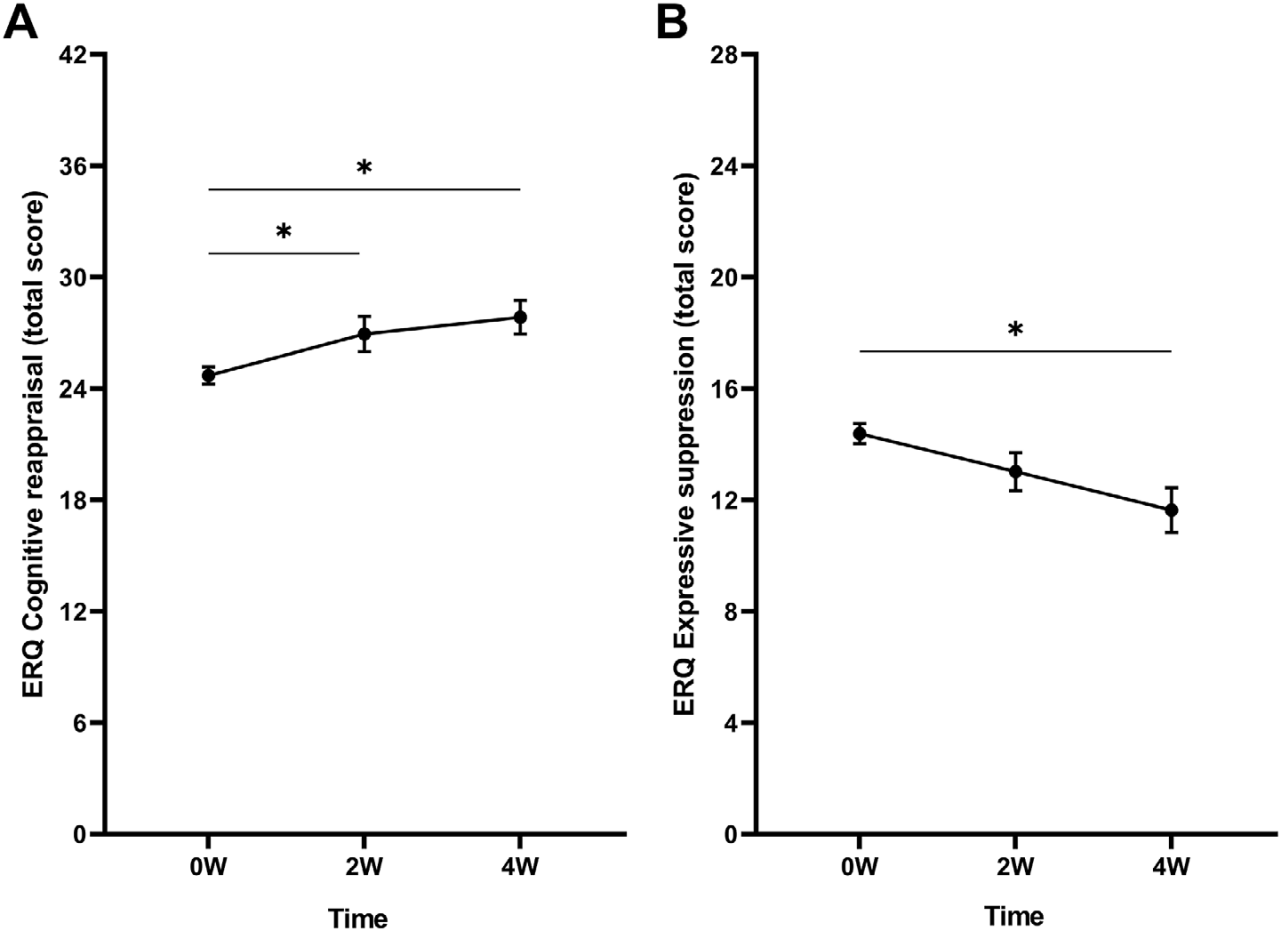
Mean raw total scores of the (A) cognitive reappraisal and (B) expressive suppression subscales of the Emotion Regulation Questionnaire (ERQ) at baseline (0 W), and the 2-week (2 W), and 4-week (4 W) time points. Error bars represent mean ± SEM. *p < .05; **p < .001.

A significant main effect of time was found (*F*_(2, 132.0)_ = 4.29, *p* = .016, *η*_p_^2^ = 0.06). Bonferroni-corrected pairwise comparisons revealed that CR scores were significantly higher at 2 W (Δ2W–0 W = 2.08, *p* = .013) and 4 W (Δ4W–0 W = 2.47, *p* = .011) compared to baseline (Figure 1A). Scores did not differ between 2 W and 4 W. Interactions between time and medication use and time and comorbidity were not significant.

#### Expressive suppression

A main effect of time was found (*F*_(2, 123.8)_ = 4.20, *p* = .017, *η*_p_^2^ = 0.06). Pairwise comparisons showed that ES scores were significantly lower at 4 W compared to baseline (Δ4W–0 W = −2.01, *p* = .002) (Figure 1B). Scores did not differ between 2 W and baseline, and 2 W and 4 W. Furthermore, no significant interactions between time and medication use and time and comorbidity were found.

### Empathy

#### Perspective-taking

**Figure 2.**
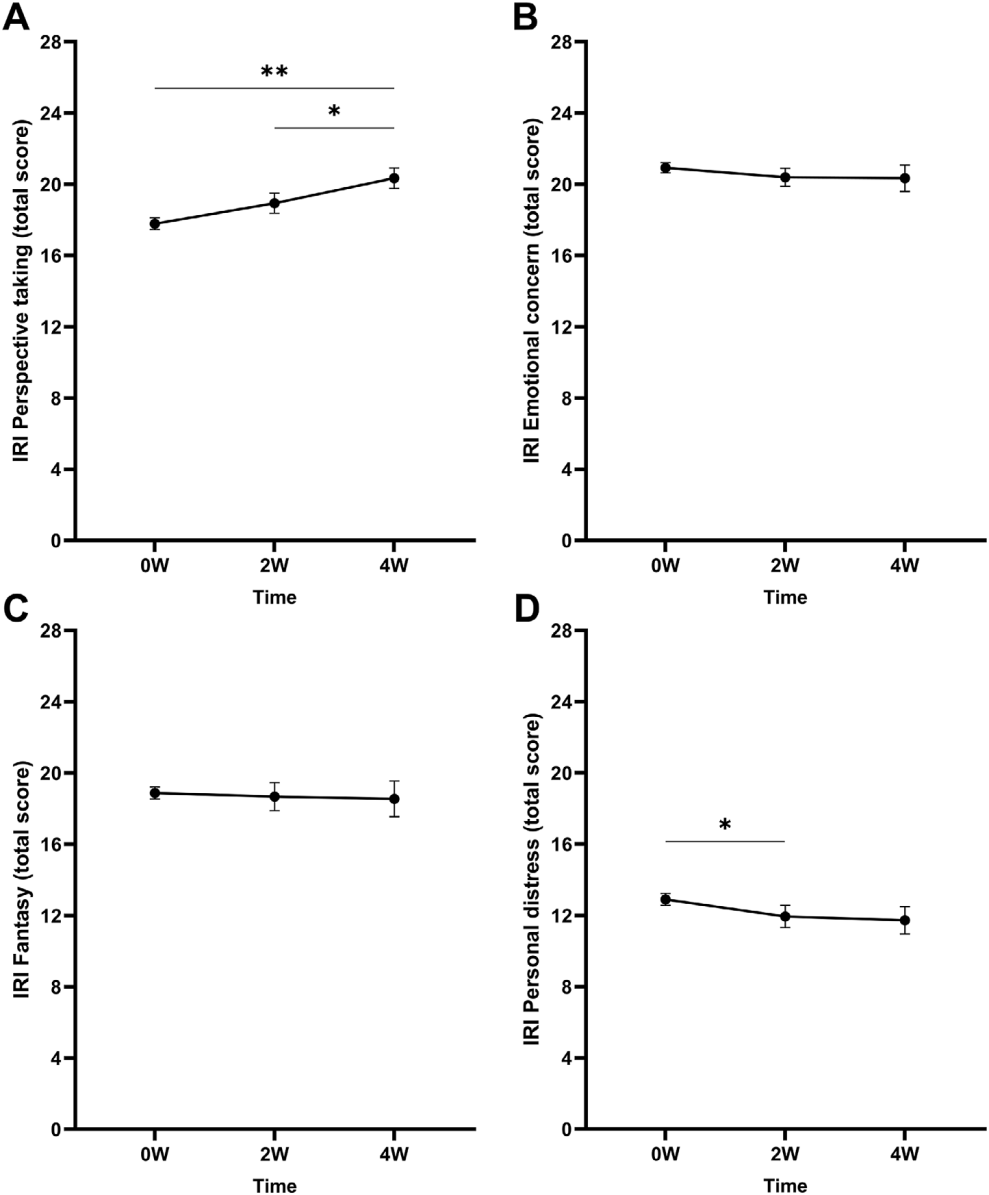
Mean raw total scores of the Interpersonal Reactivity Index (IRI) subscales (A) perspective-taking, (B) empathic concern, (C) fantasy, and (D) personal distress at baseline (0 W), and the 2-week (2 W), and 4-week (4 W) time points. Error bars represent mean ± SEM. *p < .05; **p < .001.

A main effect of time on the PT scores was found (*F*_(2, 65.9)_ = 5.01, *p* = .009, *η*_p_^2^ = 0.13). Pairwise comparisons revealed significantly higher scores at 4 W compared to baseline (Δ4W–0 W = 1.92, *p* < .001) (Figure 2A). Scores were also significantly higher at 4 W compared to 2 W (Δ4W–2 W = 1.12, *p* = .011). Scores did not differ significantly between baseline and 2 W. The interactions between time and medication use and time and comorbidity were not significant.

#### Empathic concern

The LMM did not show a main effect of time on EC scores (Figure 2B). Further, no interactions between time and medication use and time and comorbidity were found.

#### Fantasy

No significant main effect of time was found (Figure 2C). Additionally, no interactions between time and medication use and time and comorbidity were found.

#### Personal distress

A significant main effect of time was found on the PD scores (*F*_(2, 122.8)_ = 5.21, *p* = .007, *η*_p_^2^ = 0.08). Bonferroni-corrected pairwise comparisons showed that scores were lower at 2 W compared to baseline (Δ2W–0 W = −1.09, *p* = .046) (Figure 2D). No differences between the scores at baseline and 4 W and 2 W and 4 W were found. The interactions between time and medication use and time and comorbidity were nonsignificant.

## Discussion

This study aimed to investigate ER and empathy before and after 2 and 4 weeks of self-initiated MD with psychedelics in adults with ADHD or severe ADHD symptoms. Consistent with our hypothesis, self-reported ER improved post-MD, with increased CR scores at 2 and 4 weeks and decreased ES scores at 4 weeks compared to baseline. Aspects of cognitive empathy (PT) and emotional empathy (PD) were enhanced, while other aspects (fantasy and EC) remained unchanged, partially supporting our hypothesis on MD’s effects on empathy. Using conventional ADHD medication alongside MD, or having comorbid diagnoses alongside the ADHD diagnosis did not influence our findings. These results suggest that MD could benefit individuals with severe ADHD symptoms. However, Study 1’s lack of a control group raises questions about MD’s comparative effectiveness against other treatments for enhancing ER and empathy in ADHD. To address this gap, a second study was undertaken.

## Study 2

In Study 2, we included a control group and an ADHD symptom severity measure for a comprehensive understanding of MD effects on individuals with ADHD or severe ADHD symptoms. CAARS-S:SV data at the three time points were included in Study 2. Two groups were compared: individuals intending to microdose for 4 weeks without conventional ADHD medication (i.e., a subsample of Study 1) and individuals with ADHD or severe ADHD symptoms who were not MD but already using conventional ADHD medication at baseline who continued this use throughout the study (“treatment as usual” [TAU] control). We selected individuals already on this medication to ensure stable levels of ADHD symptom severity, ER, and empathy, facilitating comparison of MD-induced effects against established levels observed after stimulant use. Study 2 excluded the subset of participants from Study 1 who used conventional medication alongside MD due to small size and lower homogeneity compared to groups using MD or conventional ADHD medication exclusively.

## Methods

### Study design and participants

Study 2 replicated the design of Study 1 including the assessment moments: baseline, 2-week, and 4-week time points, without experimental manipulations. A subset of Study 1 was compared to a control group. The control group consisted of adults with diagnosed ADHD and those without a diagnosis but experiencing severe symptoms interfering with daily life, using conventional ADHD medication. ADHD symptom severity was assessed at baseline using the CAARS-S:SV [36]. Individuals without an ADHD diagnosis with T-scores lower than 65 on each subscale were excluded. All participants provided informed consent before study initiation.

### Study procedure

To recruit control group participants, both online and offline approaches were used. Online advertisements were placed on social media platforms (i.e., Reddit, Twitter, Facebook, and LinkedIn), and on www.impulsenwoortblind.nl, a Dutch ADHD advocacy organization. Paper advertisements, identical to the online advertisements, were distributed in higher education organizations like Maastricht University, and clinical ADHD facilities in Maastricht, The Netherlands. Inclusion criteria specified a minimum age of 16 years, requiring either a formal ADHD diagnosis or experiencing severe ADHD symptoms that significantly impact daily life. Information and consent procedures were identical to Study 1. Qualtrics was used as online survey platform. Responses were completely anonymous. Data were collected between September 2023 and January 2024, with approval from the Ethics Review Committee of Psychology and Neuroscience at Maastricht University (ERCPN-215_05_11_2019_A2).

### Measures

Information regarding demographics, substance use history, and psychiatric and physiological diagnoses was asked at baseline and is shown in Table 4.

**Table 4.** Demographic information collected at baseline of the microdosing only subsample of Study 1 (MED) and the conventional medication users of Study 2 (TAU)

Group		Microdosing only (MD)			Conventional medication only (TAU)		
Time point		Baseline	2-week	4-week	Baseline	2-week	4-week
*n*		180	50	38	37	27	28
Age (mean ± SD)		36.3 ± 11.0	36.1 ± 9.6	38.0 ± 8.9	37.4 ± 12.9	37.3 ± 13.8	38.9 ± 13.8
		*n* (%)	*n* (%)	*n* (%)	*n* (%)	*n* (%)	*n* (%)
Biological sex	Male	94 (52.2)	22 (44.0)	16 (42.1)	5 (13.5)	4 (14.8)	5 (17.9)
	Female	86 (47.8)	28 (56.0)	22 (57.9)	32 (86.5)	23 (85.2)	23 (82.1)
Gender	Male	90 (50.0)	20 (40.0)	14 (36.8)	5 (13.5)	4 (14.8)	5 (17.9)
	Female	86 (47.8)	28 (56.0)	22 (57.9)	32 (86.5)	23 (85.2)	23 (82.1)
	Other	4 (2.2)	2 (4.0)	2 (5.3)	–	–	–
Continent of current residence	Europe	152 (84.4)	47 (94.0)	37 (97.4)	33 (89.2)	24 (88.9)	24 (85.7)
	America (North)	22 (12.2)	2 (4.0)	1 (2.6)	3 (8.1)	2 (7.4)	3 (10.7)
	America (South)	5 (2.8)	1 (2.0)	–	–	–	–
	Asia	1 (0.6)	–	–	–	–	–
	Africa	–	–	–	–	–	–
	Antarctica	–	–	–	–	–	–
	Australia/Oceania	–	–	–	1 (2.7)	1 (3.7)	1 (3.6)
Highest level of education	Tertiary (university, trade school, college)	131 (72.8)	40 (80.0)	30 (78.9)	27 (73.0)	18 (66.7)	20 (71.4)
	Secondary (high school, academies, gymnasium, etc.)	41 (22.8)	9 (18.0)	8 (21.1)	9 (24.3)	8 (29.6)	8 (28.6)
	Primary (elementary)	8 (4.4)	1 (2.0)	–	1 (2.7)	1 (3.7)	–
Daily occupation	Computer/office work	43 (23.9)	12 (24.0)	12 (31.6)	17 (45.9) ^b^	12 (44.4) ^b^	12 (42.9) ^b^
	Studying	32 (17.8)	9 (18.0)	4 (10.5)	11 (29.7) ^b^	7 (25.9) ^b^	10 (35.7) ^b^
	Working with people	30 (16.7)	11 (22.0)	6 (15.8)	22 (59.5) ^b^	15 (55.6) ^b^	17 (60.7) ^b^
	Creative work	25 (13.9)	8 (16.0)	7(18.4)	6 (16.2) ^b^	4 (14.8) ^b^	5 (17.9) ^b^
	Physical work	10 (5.6)	3 (6.0)	3 (7.9)	8 (21.6) ^b^	5 (18.5) ^b^	6 (21.4) ^b^
	Other	40 (22.2)	7 (14.0)	6 (15.8)	6 (16.2) ^b^	6 (22.2) ^b^	5 (17.9) ^b^
Currently diagnosed^a^	Yes	113 (62.8)	35.0 (70.0)	28 (73.7)	37 (100)	27 (100)	28 (100)
	No	67 (37.2)	15 (30.0)	10 (26.3)	–	–	–
Type of disorder^b^	ADHD	106 (58.9)	31 (62.0)	25 (65.8)	37 (100)	27 (100)	28 (100)
	Depression	27 (15.0)	4 (8.0)	4 (10.5)	4 (10.8)	4 (14.8)	4 (14.3)
	Anxiety disorder	25 (13.9)	6 (12.0)	5 (13.2)	9 (24.3)	8 (29.6)	8 (28.6)
	PTSD	10 (5.6)	3 (6.0)	1 (2.6)	4 (10.8)	4 (14.8)	4 (14.3)
	Personality Disorder	13 (7.2)	3 (6.0)	1 (2.6)	–	–	–
	Dyslexia	9 (5.0)	1 (2.0)	–	2 (5.4)	2 (7.4)	2 (7.1)
	Migraines	6 (3.3)	3 (6.0)	3 (7.9)	4 (10.8)	3 (11.1)	4 (14.3)
	Chronic pain	5 (2.8)	2 (4.0)	2 (5.3)	–	–	–
	Cluster headaches	2 (1.1)	2 (4.0)	2 (5.3)	–	–	–
	Substance use disorder	1 (0.6)	1 (2.0)	–	1 (2.7)	1 (3.7)	1 (3.6)
	Autism/Asperger syndrome	3 (1.7)	1 (2.0)	–	2 (5.4)	2 (7.4)	2 (7.1)
	OCD	2 (1.1)	1 (2.0)	–	1 (2.7)	–	1 (3.6)
	Bipolar disorder	3 (1.7)	–	–	–	–	–
	Schizophrenia	–	–	–	–	–	–
	Other	12 (6.7)	3 (6.0)	2 (5.3)	2 (5.4)	2 (7.4)	2 (7.1)
Experience with psychedelic drug^c^	Yes	148 (82.2)	38 (76.0)	30 (78.9)	8 (21.6)	5 (18.5)	6 (21.4)
	No	32 (17.8)	12 (24.0)	8 (21.1)	29 (78.4)	22 (81.5)	22 (78.6)

Absolute and relative frequencies are shown. Numbers in parentheses indicate the percentage corresponding to the absolute frequencies.

a Are you currently diagnosed by a medical doctor or therapist with a psychiatric, neurological, or physical disorder?

b Numbers do not add up to the sample size, because multiple answers were possible.

c Do you have experience with at least one of the following psychedelics? Ayahuasca, DMT, 5-MeO-DMT, LSD, novel lysergamides (e.g., 1P-LSD, ALD-52), psilocybin/psilocin (magic mushrooms, truffles), *salvia divinorum*, ibogaine, mescaline (e.g., san pedro, peyote).

#### Medication type, dose, and use of psychedelics

At both the 2- and 4-week time points, participants in the TAU group were asked if they had taken ADHD medication within the past 2 weeks, and if so, what type and dose they used (Table 5). Similarly, participants in the TAU group were asked at the 2- and 4-week time points if they had taken any psychedelic substances in the past 2 weeks. If this was the case, they were excluded from the analyses.

**Table 5.** ADHD-related information collected at baseline from individuals who had been diagnosed with ADHD in the past from the microdosing only group (MD; *n* = 106) and the conventional medication only group (TAU; *n* = 37)

Group		Microdosing only (MD)	Conventional medication only (TAU)
		*n* (% of 106)	*n* (% of 37)
Age of receiving ADHD diagnosis	Younger than 10 years old	5 (4.7)	1 (2.7)
	10–14 years old	5 (4.7)	1 (2.7)
	15–19 years old	12 (11.3)	6 (16.2)
	20–29 years old	43 (40.6)	7 (18.9)
	30–39 years old	29 (27.4)	11 (29.7)
	40–49 years old	8 (7.5)	8 (21.6)
	Over 50 years old	4 (3.8)	3 (8.1)
Current ADHD medication use	No, never	22 (20.8)	–
	Used to, but not anymore	84 (79.2)	–
	Reasons for discontinuing	*n* (% of 84)	
	It did not relieve my symptoms	17 (20.2)	–
	Because of psychological side-effects	51 (60.7)	–
	Because of physical side-effects	53 (63.1)	–
	I do not want to mention	1 (1.2)	–
	Other reasons	21 (25.0)	–
	Yes, I am currently using these	–	37 (100)
	Medication types	–	*n* (% of 37)
	Amphetamine^a^	–	17 (45.9)
	Methylphenidate	–	16 (43.2)
	Other	–	4 (10.8)

a Dexamphetamine and Lisdexamfetamine fell under the category amphetamine.

#### ADHD symptoms, ER, and empathy

ADHD symptoms, ER, and empathy were assessed at baseline and the 2- and 4-week time points with the CAARS-S:SV [36], ERQ [6], and the IRI [38].

### Statistical analyses

Data collected from the TAU group were pooled with the data from the MD-only subsample of Study 1 (MD). Demographic variables, information regarding diagnoses, and medication types and doses used during the study were described using descriptive statistics. Similar analyses were used as in Study 1 (i.e., LMM) to compare the MD and TAU groups regarding ADHD symptoms, ER, and empathy at baseline (0 W), and the 2- (2 W) and 4-week (4 W) time points. Each LMM contained the within-subjects factor time (three levels: 0 W, 2 W, and 4 W) and the between-subjects factor group (MD vs. TAU). The fixed part consisted of time, group, and the interaction term between time and group. Comorbidity (yes/no) was included as covariate, but removed from the model if nonsignificant. Only the time by group interaction was interpreted in case of significance; otherwise, the main effect was interpreted if applicable.

To compare the changes in ADHD symptom severity between MD and TAU, the CAARS-S:SV subscale T-scores (inattention, hyperactivity/impulsivity, DSM-IV total symptom score, ADHD index) were analyzed in separate LMMs. To compare the changes in ER between MD and TAU, ERQ subscale scores (CR and ES) were analyzed in separate LMMs. To compare the changes in emotional and cognitive empathy between the MD and TAU groups, the IRI subscales (PT, F, EC, PD) were included as dependent variables in separate LMMs.

The best-fitting covariance structure was chosen based on the lowest AIC value. REML estimation was used to estimate missing data. Simple effects and pairwise comparisons followed from a significant interaction and main effects, respectively, and were corrected for multiple comparisons using Bonferroni correction. Similarly to Study 1, a significance level of. 025 was used. Effect sizes were described using partial eta squared (*η*_p_^2^) values, considering 0.01, 0.09, and 0.25 as small, medium, and large, respectively [40]. Only significant effects will be described.

## Results

Over half (*n* = 42; 53.8%) of the initial survey respondents (*n* = 78) were using conventional ADHD medication at baseline. One participant had used a psychedelic substance during the study, and four participants did not complete the ERQ and IRI and were therefore excluded. All 37 remaining participants had ADHD diagnoses so none were excluded based on the CAARS-S:SV. Sample sizes for further analyses were 180, 50, 38 for the MD group (subsample of Study 1) and 37, 27, 28 for the TAU group at 0 W, 2 W, and 4 W, respectively. Tables 4 and 5 present demographic information for both groups at the three time points and ADHD-related information for the ADHD-diagnosed subsample, respectively. Table 6 provides details on conventional medication types and doses of the TAU group during the study. Comorbidity was not significant in any LMM analyses and was removed from all models.

**Table 6.** Conventional ADHD medication type and dose used by the conventional medication group (TAU) in the past 2 weeks assessed at the 2- (2 W) and 4-week (4 W) time points

Time point (sample size)	2 W (*n* = 27)		4 W (*n* = 28)	
	Frequency (% of 27)	Mean dose (Min–Max; SD)	Frequency (% of 28)	Mean dose (Min–Max; SD)
Methylphenidate	11 (41.7)	33.6 mg (5–72; 20.8)	12 (42.9)	44.3 mg (10–80; 22.2)
Amphetamine	–	–	1 (3.6)	10 mg (−)
Dexamphetamine	7 (25.9)	21.8 mg (8–40; 11.9)	5 (17.9)	36.0 mg (5–90; 33.8)
Lisdexamfetamine	6 (22.2)	65 mg (20–150; 44.6)	7 (25.0)	58.6 mg (20–130; 36.7)
Atomoxetine hydrochloride	1 (3.7)	40 mg (−)	1 (3.6)	40 mg (−)
Guanfacine	1 (3.7)	4 mg (−)	1 (3.6)	4 mg (−)
Other: moclobemide	1 (3.7)	600 mg (−)	1 (3.6)	600 mg (−)

### ADHD symptom severity

#### Inattention

**Figure 3.**
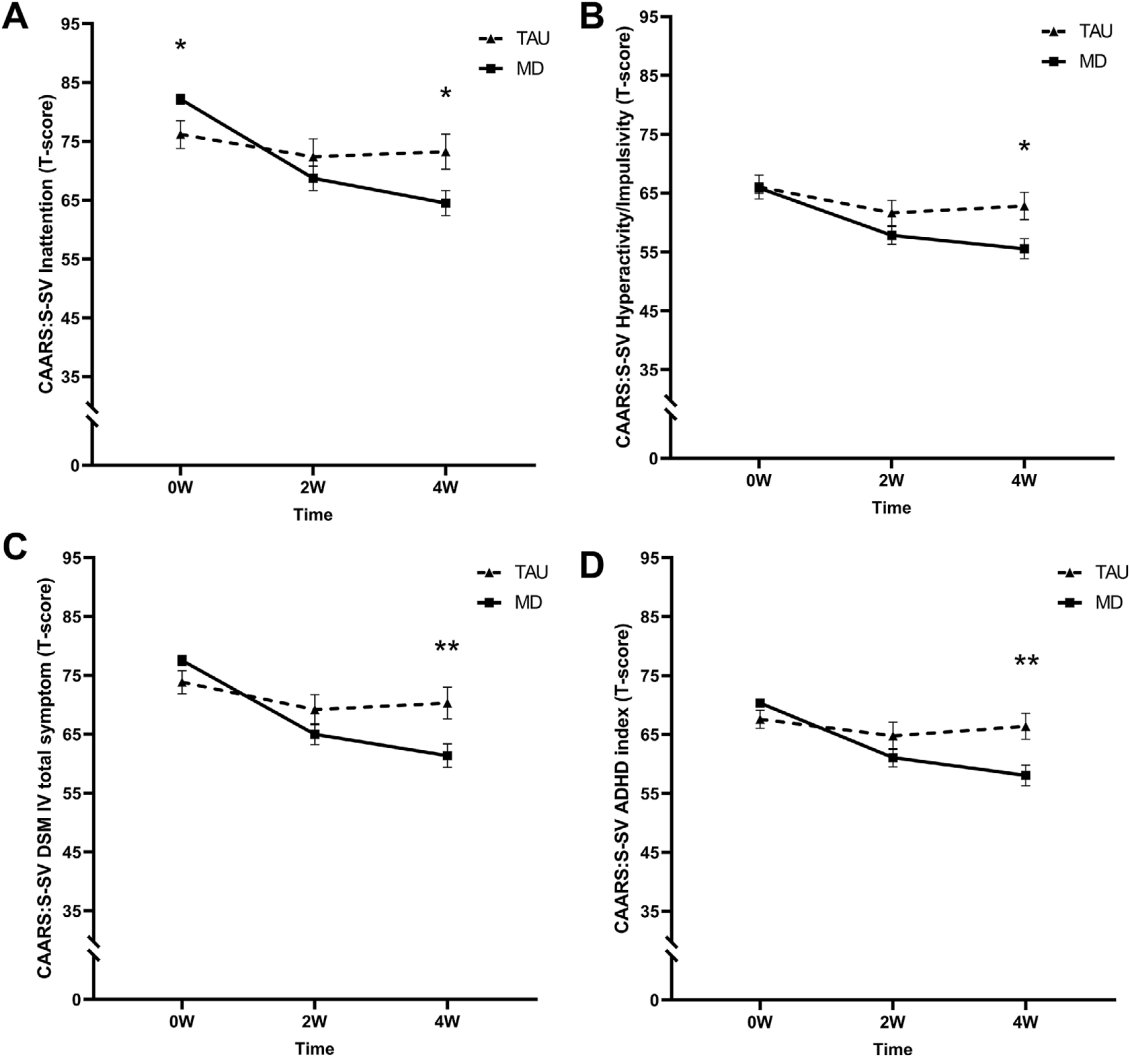
Mean T-scores of the short screening version of the Conners’ Adult ADHD Rating Scale (CAARS-S:SV) subscales (A) inattention, (B) hyperactivity/impulsivity, (C) DSM-IV total symptoms, and (D) ADHD index at baseline (0 W), and the 2-week (2 W), and 4-week (4 W) time points. The solid line represents the microdosing group (MD), and the dotted line represents the medication group (TAU). A significant time by group interaction was found on all CAARS-S:SV subscales. Asterisks (*) indicate the time points where the groups differed significantly. Error bars represent mean ± SEM. *p < .05; **p < .001.

A significant time by group interaction (*F*_(2, 85.8)_ = 10.26, *p* < .001, *η*_p_^2^ = 0.19) revealed that the MD group had a higher mean T-score at baseline but lower at 4 W compared to the TAU group (see Figure 3A).

#### Hyperactivity/impulsivity

A significant time by group interaction was found (*F*_(2, 150.5)_ = 4.81, *p* = .000, *η*_p_^2^ = 0.06). At 4 W, T-scores were lower for the MD compared to the TAU group (see Figure 3B).

#### DSM-IV total symptoms

A significant time by group interaction was found (*F*_(2, 173.5)_ = 9.55, *p <* .001, *η*_p_^2^ = 0.10). At 4 W, T-scores were lower for the MD compared to the TAU group (see Figure 3C).

#### ADHD index

The LMM revealed a significant time by group interaction (*F*_(2, 70.6)_ = 17.03 *p <* .001, *η*_p_^2^ = 0.33). At 4 W, T-scores were lower for the MD compared to the TAU group (see Figure 3D).

### Emotion regulation

#### Cognitive reappraisal

**Figure 4.**
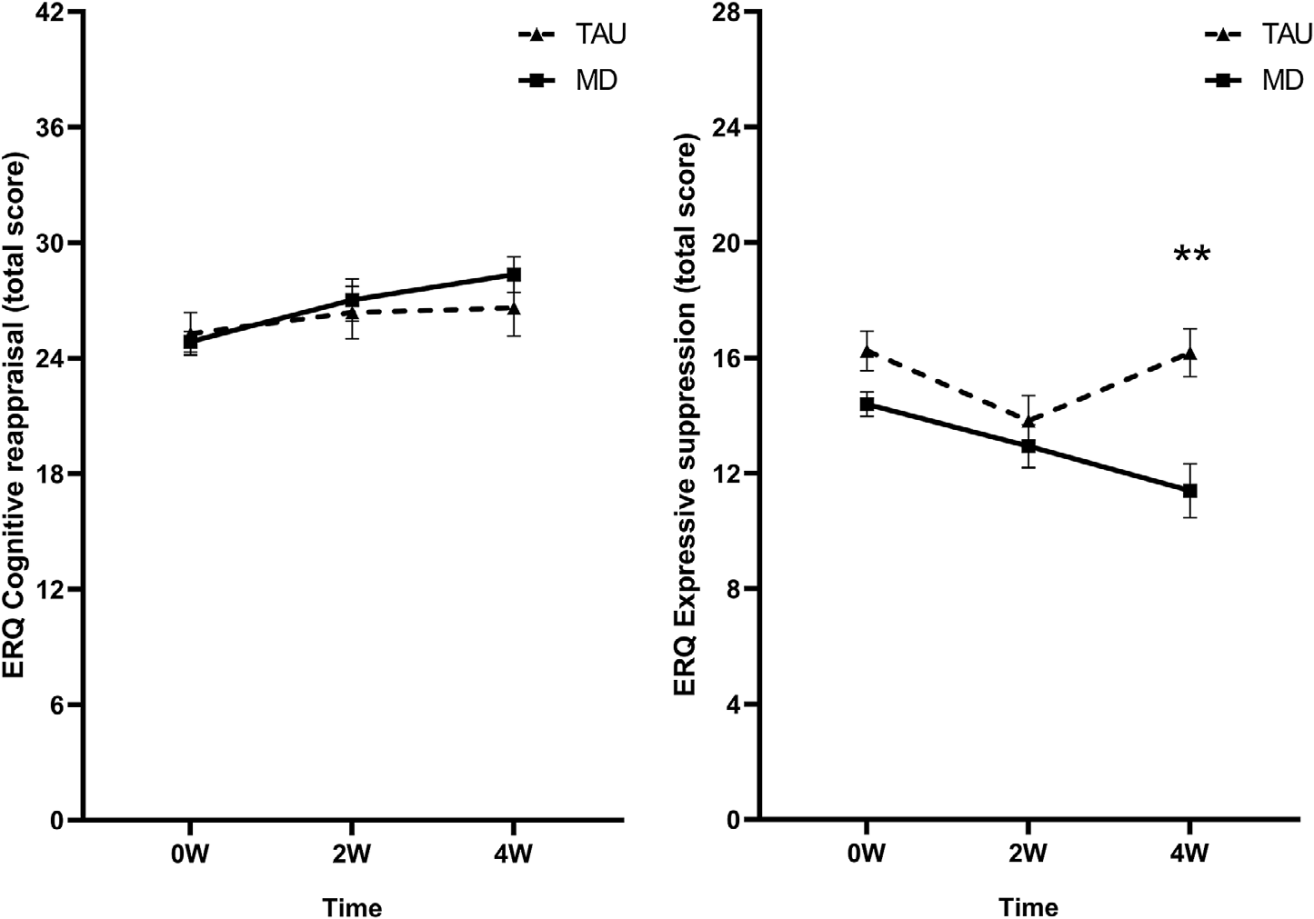
Mean raw total scores of the (A) cognitive reappraisal and (B) expressive suppression subscales of the Emotion Regulation Questionnaire (ERQ) at baseline (0 W), and the 2-week (2 W), and 4-week (4 W) time points. The solid line represents the microdosing group (MD), and the dotted line represents the medication group (TAU). A significant time by group interaction was found on the mean expressive suppression scores. Asterisks (*) indicate the time points where the groups differed significantly. Error bars represent mean ± SEM. *p < .05; **p < .001.

No significant time by group interaction was found (*F*_(2, 152.9)_ = 1.24 *p =* .294, *η*_p_^2^ = 0.02). Only a main effect of time was found (*F*_(2, 152.9)_ = 3.29 *p =* .040, *η*_p_^2^ = 0.04), though pairwise comparisons were nonsignificant (see Figure 4A).

#### Expressive suppression

A significant time by group interaction was found (*F*_(2, 146.6)_ = 4.58 *p =* .012, *η*_p_^2^ = 0.06), showing that mean ES scored were lower for the MD group compared to the TAU group at 4 W (see Figure 4B).

## Empathy

### Cognitive empathy

#### Perspective-taking

**Figure 5.**
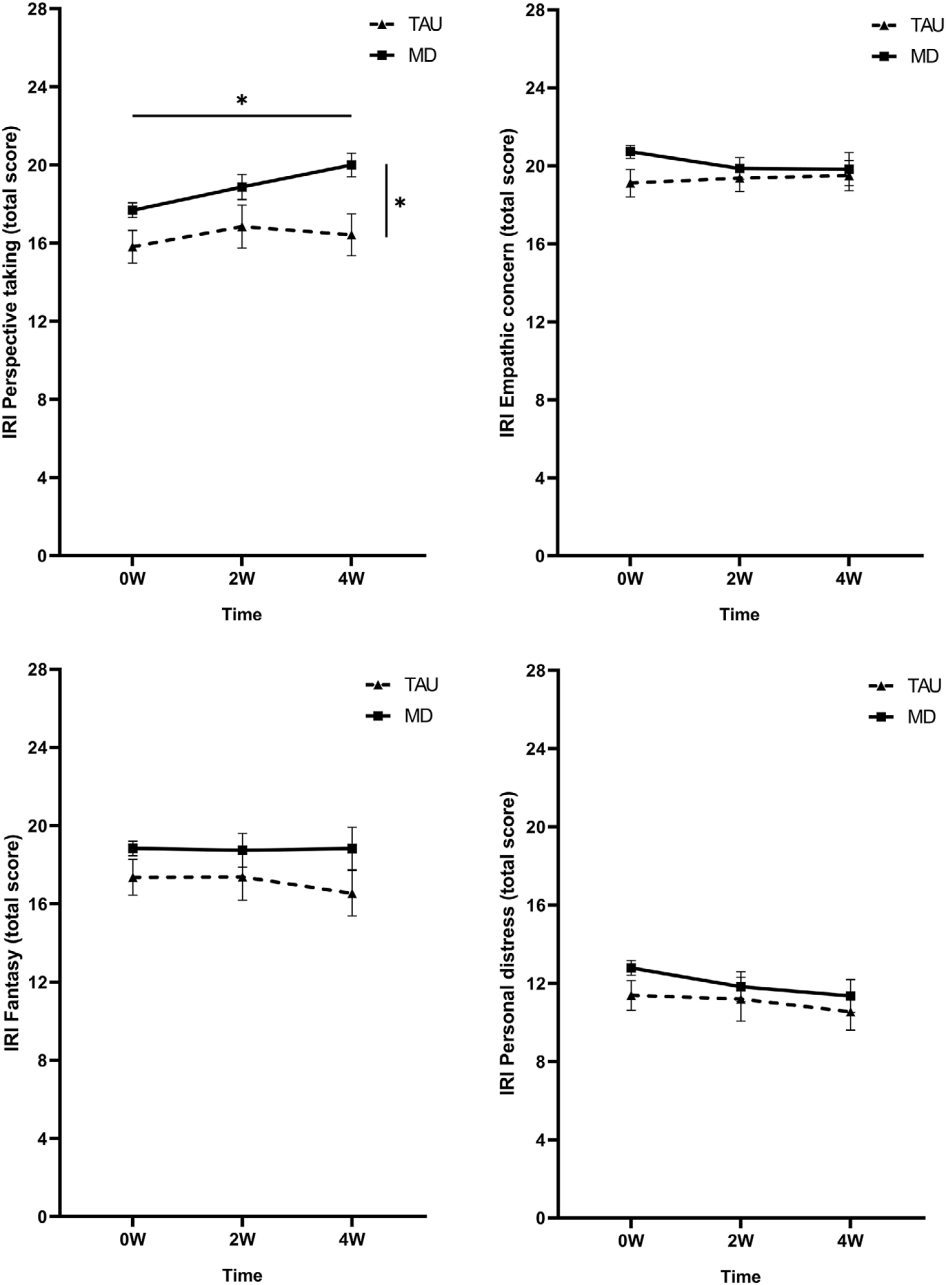
Mean raw total scores of the Interpersonal Reactivity Index (IRI) subscales (A) perspective-taking, (B) empathic concern, (C) fantasy, and (D) personal distress at baseline (0 W), and the 2-week (2 W), and 4-week (4 W) time points. The solid line represents the microdosing group (MD), and the dotted line represents the medication group (TAU). Significant main effects of time and group were found on the mean perspective-taking scores. The horizontal line with asterisk (*) represents the Time effect, whereas the vertical line with asterisk (*) represents the group effect. Error bars represent mean ± SEM. *p < .05; **p < .001.

No significant time by group interaction was found (*F*_(2, 69.7)_ = 1.14 *p =* .325, *η*_p_^2^ = 0.03). However, a time effect was found (*F*_(2, 69.7)_ = 3.88 *p =* .025, *η*_p_^2^ = 0.10). Corrected pairwise comparisons revealed higher scores at 4 W compared to 0 W (Δ4W–0 W = 1.16, *p* = .020) (see Figure 5A). Additionally, a main effect of group was found (*F*_(1, 147.5)_ = 6.97 *p =* .009, *η*_p_^2^ = 0.09), with generally higher scores for the MD group compared to the TAU group (ΔMD-TAU = 2.32, *p* = .009).

#### Fantasy

No time by group interaction (*F*_(2, 100.6)_ = 0.66 *p =* .518, *η*_p_^2^ = 0.01), main time (*F*_(2 100.6)_ = 2.77 *p =* .067, *η*_p_^2^ = 0.05), or main group effect (*F*_(1 152.2)_ = 1.83 *p =* .178, *η*_p_^2^ = 0.02) was found (see Figure 5C).

### Emotional empathy

#### Empathic concern

No time by group interaction (*F*_(2, 101.4)_ = 1.07 *p =* .346, *η*_p_^2^ = 0.02), main time (*F*_(2, 101.4)_ = 0.49 *p =* .612, *η*_p_^2^ = 0.01), or main group effect (*F*_(1, 136.7)_ = 1.78 *p =* .185, *η*_p_^2^ = 0.03) was found (see Figure 5B).

#### Personal distress

No time by group interaction (*F*_(2, 101.6)_ = 1.16 *p =* .319, *η*_p_^2^ = 0.02), main time (*F*_(2, 101.6)_ = 1.70 *p =* .187, *η*_p_^2^ = 0.03), or main group effect (*F*_(1, 131.5)_ = 0.37 *p =* .542, *η*_p_^2^ = 0.01) was found (see Figure 5D).

## General discussion

These studies assessed the effects of MD on ER and empathy in adults with severe ADHD symptoms. Study 1 found improvements in ER and some aspects of cognitive and emotional empathy. Study 2 introduced a control group (treatment-as-usual), and measured ADHD symptom severity, providing a broader understanding of MD effects. Results indicated that adults with severe ADHD symptoms experienced reduced ADHD symptoms through MD, evidenced by lower symptom severity after 4 weeks compared to conventional medication users. At baseline, the MD sample scored higher on the ADHD inattention subscale, as expected due to the control group’s medication use at baseline. No other subscales differed at baseline between the groups. After 4 weeks, the MD group had lower ADHD symptom severity scores on all subscales compared to the TAU group. Additionally, the MD group scored below the clinically elevated symptoms threshold on all subscales, while the medication group scored below this threshold on only one subscale. These findings suggest MD could effectively decrease ADHD symptoms, consistent with previous survey studies indicating its potential therapeutic effect on ADHD [21, 23].

However, evidence supporting positive effects on ER and empathy was relatively weaker. Specifically, ES, the ER process involving inhibiting ongoing emotion-expressive behavior, was the only aspect positively influenced by MD compared to individuals consistently using conventional medication. ES of the MD group was at baseline similar on average to mean scores of an ADHD sample (*n* = 30; of which nine used ADHD medication) [41], and several normative samples [6, 37, 42]. While the level of ES unexpectedly dropped for the TAU group after 2 weeks and went back to the baseline level 2 weeks later, ES in the MD sample steadily decreased over 4 weeks, reaching mean scores similar to a sample of healthy adults [41]. ES is an ER process employed at a late stage in the emotion-generative process [5, 43]. It requires constant management of emotional responses, potentially depleting cognitive resources and compromising social functioning [44]. Further, it has been related to negative self-feelings, inauthenticity, and depressive symptoms [6]. Previous research suggested that using ES serves as a compensatory mechanism for ER in ADHD [45]. Therefore, the finding that MD reduced ES may indicate improved ER abilities in the MD sample. This finding is notable as conventional pharmacological ADHD treatments have limited effects on ER abilities [16–18], which was reflected by the ES scores reported by the control group, as they were similar or somewhat higher at baseline and 4-weeks compared to another ADHD sample [41] and general population samples [6, 37, 42]. The finding that MD induced positive effects on ADHD symptoms and ER is interesting. Psychedelics primarily target the serotonin (5-HT) 2A receptor [46], which is highly expressed across the neocortex, including the prefrontal cortex (PFC), which is involved in executive functions and attention regulation [47]. The 5-HT2A receptor modulates PFC activity, which inhibits amygdala activity for successful ER [48]. Studies have indicated hyperactivity within the amygdala in ADHD [5] and reduced functional connectivity between the PFC and amygdala, suggesting compromised inhibitory regulation by the PFC, which was related to ES in ADHD [45]. MD may modulate PFC activity to increase inhibitory activity on the amygdala thereby improving ER.

The MD-induced increase in CR, that is, the ability to intentionally reinterpret an emotion-eliciting event, found in Study 1 was a weak effect (*η*_p_^2^ = 0.06) that disappeared when comparing individuals who were solely MD to individuals using conventional medication. Further, MD showed no effects on emotional and cognitive empathy. The increase in PT in Study 1 seemed to be a general trend, with both groups increasing over time. However, the MD sample consistently scored higher on PT compared to the medication group. Overall, the MD group scored high on average at baseline on all empathy subscales compared to neurotypical adults [49–51], potentially indicating a ceiling effect or lack of sensitivity to MD effects. Further, Study 1 found a decrease in PD scores, which disappeared when including the control group. High PD levels, associated with poor social functioning and feelings of discomfort in socially tense situations [39], remained unchanged after MD.

Overall, the positive changes in sociability seen in previous MD studies [22, 26–28] may stem from improvements in ER, ES specifically, or other aspects of sociability not captured here (e.g., communication skills, social connectedness). The limited evidence of positive effects of MD on sociability could also be because these effects are possibly less evident in individuals with severe ADHD symptoms.

The study’s prospective naturalistic design is a key strength, allowing a comparison of scores before and after MD. Focusing on ER and empathy extends the investigation beyond core ADHD symptoms, addressing broader problem features. Furthermore, given the transdiagnostic properties of ER and empathy [52, 53], MD may hold potential as a treatment for various patient populations experiencing ER difficulties, such as borderline [54, 55] and narcissistic personality disorders [56, 57], social anxiety disorder [58, 59], autism spectrum disorder [60, 61], and mood disorders [58, 62]. For MD to be considered a treatment option, it should be viewed within an integrated framework, guided by a trained therapist or clinician, who incorporates MD into existing treatment modalities.

Most limitations of this study are inherent in the naturalistic design. First, participant self-selection and potential dropouts due to dissatisfaction with MD may have introduced bias. Lack of experimental control over factors such as drug type, dose, route of administration, and storage conditions, could have impacted the effects observed. Contextual factors, like intentions, expectations, and physical, social, and cultural factors, were not assessed, potentially impacting the observed MD-effects [63]. Further, the study only used self-report measures; while this provides valuable subjective information, future (controlled) studies should include behavioral measures too as they are less susceptible to response biases. Further, the diagnostic status of the participants was based on self-reports and therefore uncertain. Future randomized controlled trials could easily address this by involving an independent expert trained in using various assessment tools including a semi-structured interview to screen participants prior to enrolment. Finally, we did not follow-up on future psychedelic or substance use. However, preclinical studies suggest minimal, if any, potential for abuse [46, 64], with some studies exploring psychedelics as addiction treatment [65], although caution is still warranted.

To conclude, this study found positive effects of 4 weeks of MD on ADHD symptoms and ER in individuals with severe ADHD symptoms compared to those using conventional ADHD medication. No effects on empathy were found. Further research is required through placebo-controlled studies to determine if the effects of MD on ADHD are genuine and not solely due to the placebo effect.
